# Differential Cortical c-Fos and Zif-268 Expression after Object and Spatial Memory Processing in a Standard or Episodic-Like Object Recognition Task

**DOI:** 10.3389/fnbeh.2013.00112

**Published:** 2013-08-22

**Authors:** Flávio Freitas Barbosa, José Ronaldo Santos, Ywlliane S. Rodrigues Meurer, Priscila Tavares Macêdo, Luane M. Stamatto Ferreira, Isabella M. Oliveira Pontes, Alessandra Mussi Ribeiro, Regina Helena Silva

**Affiliations:** ^1^Memory and Cognition Studies Laboratory, Department of Psychology, Federal University of Paraíba, João Pessoa, Brazil; ^2^Laboratory of Behavioral Neurobiology, Department of Biology, Federal University of Sergipe, São Cristóvão, Brazil; ^3^Memory Studies Laboratory, Department of Physiology, Federal University of Rio Grande do Norte, Natal, Brazil

**Keywords:** recognition memory, spatial memory, episodic memory, immediate early genes, plasticity, hippocampus

## Abstract

Episodic memory reflects the capacity to recollect what, where, and when a specific event happened in an integrative manner. Animal studies have suggested that the medial temporal lobe and the medial pre-frontal cortex are important for episodic-like memory (ELM) formation. The goal of present study was to evaluate whether there are different patterns of expression of the immediate early genes c-Fos and Zif-268 in these cortical areas after rats are exposed to object recognition (OR) tasks with different cognitive demands. Male rats were randomly assigned to five groups: home cage control, empty open field (CTR-OF), open field with one object (CTR-OF + Obj), novel OR task, and ELM task and were killed 1 h after the last behavioral procedure. Rats were able to discriminate the objects in the OR task. In the ELM task, rats showed spatial (but not temporal) discrimination of the objects. We found an increase in the c-Fos expression in the dorsal dentate gyrus (DG) and in the perirhinal cortex (PRh) in the OR and ELM groups. The OR group also presented an increase of c-Fos expression in the medial prefrontal cortex (mPFC). Additionally, the OR and ELM groups had increased expression of Zif-268 in the mPFC. Moreover, Zif-268 was increased in the dorsal CA1 and PRh only in the ELM group. In conclusion, the pattern of activation was different in tasks with different cognitive demands. Accordingly, correlation tests suggest the engagement of different neural networks in the tasks used. Specifically, perirhinal-DG co-activation was detected after the what-where memory retrieval, but not after the novel OR task. Both regions correlated with the respective behavioral outcome. These findings can be helpful in the understanding of the neural networks underlying memory tasks with different cognitive demands.

## Introduction

Human episodic memory refers to our capacity to recall when and where a specific event (what) happened (Tulving, 2001, 2002; Dere et al., 2006). Some researchers have pointed out that it is a unique human capability, since only humans have autonoetic awareness (Tulving, 2002; Clayton et al., 2003; Dere et al., 2006). However, recently, researchers have found that other animals can also recollect what-where-when an episode occurred. Clayton et al. (2003) distinguished between the phenomenological criteria and the behavioral criteria and called this non-human memory system episodic-like memory (ELM). Some authors also described that animals can use these memories in an integrative manner, a fundamental issue in the episodic memory definition (Clayton et al., 2003; Dere et al., 2006; Kart-Teke et al., 2006). In this context, object recognition (OR) tasks have been used to accesses ELM in rodents.

The novel OR task accesses the capacity of rats in discriminating new objects from old ones in a familiar arena, being an important tool to investigate the “what” aspect of the ELM. Although hippocampal function is essential to human episodic memory (Squire and Zola, 1996; Tulving, 2002), the results regarding the role of this structure in the OR in rodents are controversial (Brown and Aggleton, 2001; Aggleton and Brown, 2006; Ainge et al., 2006). Conversely, lesions (Barker et al., 2007), temporary inactivation (Winters and Bussey, 2005b), or NMDA blockade in the perirhinal cortex (PRh) (Winters and Bussey, 2005a; Barker and Warburton, 2008) results in impairment of novel OR performance.

Variations of the OR task have been developed to study the spatial and temporal aspects of the ELM as well (Dere et al., 2007; Hoge and Kesner, 2007). Dere et al. (Dere et al., 2005a,b; Kart-Teke et al., 2006) developed an OR task in which mice or rats have to discriminate when and where they previously encountered a specific familiar object. Recently, we have adapted this protocol using a 24-h retention delay (Barbosa et al., 2010), in order to study separately the acquisition, consolidation, and retrieval mnemonic processes. The use of this retention delay allows pharmacological manipulations, as well as the investigation of immediate early genes expression related to the ELM components.

Evaluation of immediate-early genes (IEGs) has been used to explore how different neural regions are recruited after a behavioral stimulation (Guzowski et al., 2004; Kubik et al., 2007). c-Fos protein is one of the most common markers of neuronal plasticity used in the field. Studies have described an increase in the expression of c-Fos in the PRh after rats were exposed to new visual stimuli, but not familiar ones (Wan et al., 1999, 2001). Interestingly, similar increases were not found in the hippocampus (HP) (Wan et al., 1999, 2001; Aggleton et al., 2012), which is in agreement with some lesion studies (Dix and Aggleton, 1999; Barker and Warburton, 2011). However, when rats were allowed to explore new or familiar objects (instead of single visual exposition to new or familiar stimuli) an increase in the c-Fos expression in the hippocampal subfields was reported (Albasser et al., 2010, 2013). Thus, actively exploring objects in a familiar arena engage hippocampal activity, although this region might not be essential in this task because lesions in this area do not elicit deficits (Mumby et al., 2002; Hoge and Kesner, 2007).

On the other hand, the engagement of the HP has been reported when spatial and/or temporal components are involved in the recognition task (Mumby et al., 2002; Hoge and Kesner, 2007; Barker and Warburton, 2011; Barbosa et al., 2012). In this respect, Castilla-Ortega et al. (2012) studied c-Fos activation after an ELM task in wild-type mice and LPA_1_-null mice. In the task used in that study, the animals were supposed to discriminate between old and recent objects (temporal order) as well as the old-displaced and the old-stationary object (spatial memory). However, wild-type mice showed only what-when memory, which was impaired in the LPA_1_-null mice. They found an increase in c-Fos expression in the dentate gyrus (DG), CA1 subregion, and in the medial prefrontal cortex (mPFC) in the normal mice. Unfortunately, this previous study included only a home cage control (CTR-HC) group, which limits the interpretation of the findings. Indeed, it has been demonstrated that environmental novelty *per se* can induce increase in c-Fos expression in the hippocampal formation (Jenkins et al., 2004; VanElzakker et al., 2008).

While c-Fos studies provide information regarding brain areas activation after a certain event, the IEG Zif-268 has been implicated in long-term memory consolidation (Bozon et al., 2002). Zif-268 knock-down mice are impaired in different spatial and non-spatial learning tasks, as well as in the expression of late LTP (Davis et al., 2000; Jones et al., 2001). Jones et al. (2001) showed that mutant mice lacking zif-268 gene were able to express early LTP in the DG, but not late LTP (after 24 or 48 h). Zif-268 has also been implicated in the novel OR and in object location tasks when there was a long interval between training and test (Bozon et al., 2003b). However, these studies did not evaluate the pattern of this IEG in different neural substrates. More recently, Soulé et al. (2008) found that object-in-place task induces an increase in *zif-268* in the rat DG. To our knowledge there are no studies addressing the pattern of Zif-268 expression in different cortices after variations of OR tasks (with different cognitive demands).

The goal of present study was to evaluate whether there are different patterns of expression of the immediate early genes c-Fos and Zif-268 in the medial temporal lobe structures and mPFC after animals are exposed to OR tasks with distinct cognitive demands. Although both IEGs are approached as plasticity markers, their co-activation in the same neural regions is not unequivocal (Herdegen and Leah, 1998; Bernabeu et al., 2006). We used the novel OR task and an ELM task. In the first task rats had to discriminate between new and familiar objects, while in the second animals had to discriminate familiar objects spatiotemporally. It is expected that different neural regions are engaged in this two recognition tasks. For example, while HP and mPFC are essential to spatiotemporal processing, they do not seem to be involved in the novel item recognition process (Hoge and Kesner, 2007; DeVito and Eichenbaum, 2010; Aggleton et al., 2012). In order to verify the specificity of the results of IEGs expression, we added not only CTR-HCs but also rats exposed to an empty open-field or to an open-field with one novel object.

## Materials and Methods

### Animals

Thirty-nine 3-month old male Wistar rats (250–400 g) were housed under controlled temperature (25 ± 1°C) and a 12/12 h light/dark cycle (lights on at 6.30 a.m.). Food and water were offered *ad libitum*. All animals were handled for 10 min/day for 5 days before the experiments start. The rats were handled accordingly to Brazilian law for the use of animals in scientific research (Law Number 11.794) and all procedures were approved by the local ethics committee (protocol number 049/2012). All efforts were made to minimize animal pain, suffering, or discomfort as well as the number of rats used.

### Apparatus and objects

The behavioral tests were conducted in a circular open-field (84 cm in diameter surrounded by a 32-cm height wall), made of wood and painted in black. There were external visual cues in the room that rats could use for spatial learning. Three sets of objects (made of plastic and filled with cement to ensure that animals would not displace them) were used in a random manner among the experiments. The objects used included a sugar bowl, a mug, and a goblet. They differed in height (9–12 cm), width (6–10 cm), color, and shape. The apparatus and objects were cleaned with a 5% alcohol/water solution after each behavioral session. A previous experiment with other rats demonstrated no spontaneous preference for any of these objects. The sessions were recorded by a digital camera placed above the apparatus. The behavioral parameters were registered by an animal tracking software (Anymaze, Stoelting, USA).

### Experimental design

**Figure 1:**
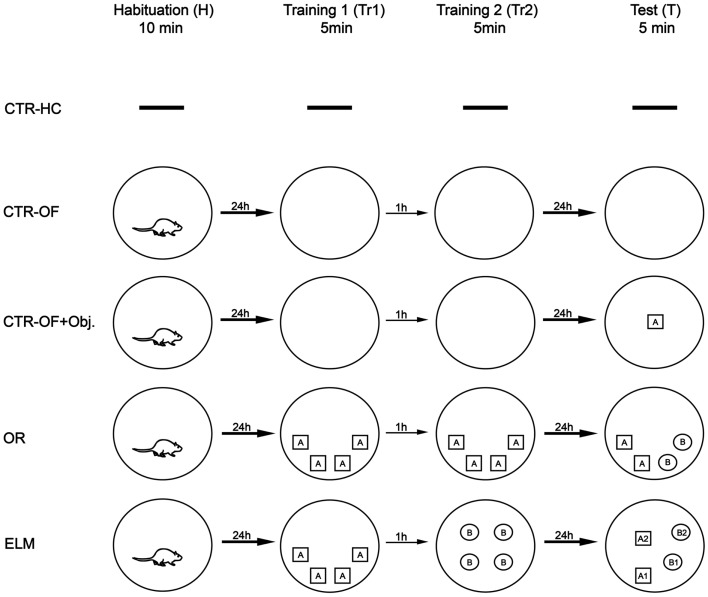
**Experimental procedure**. CTR-HC, home cage control (without behavioral sessions); CTR-OF, open field control (four expositions to the empty open field); CTR-OF + Obj, open field + object control (exposition to an object in the last behavioral session); OR, object recognition task (discrimination between novel and familiar objects); ELM, episodic-like memory task (spatiotemporal discrimination of familiar objects).

Experimental design is schematized in Figure 1. The animals were divided into five groups: CTR-HC (*n* = 8), open-field control (*n* = 8), open-field + object control (*n* = 6), OR task (*n* = 8), and ELM task (*n* = 9). Twenty-four hours prior to the beginning of the tasks, all animals underwent a 10-min habituation session in the open field, except for the CTR-HC group. Each session was performed in an interval of 24 h, except for the two training sessions that were performed with an interval of 1 h between them. The behavioral procedures for each group were the following:

(1) CTR-HC: on the sixth day after the handling, the animals were removed from their home cages and euthanized without apparatus exposure. The goal of this group was to measure IEGs basal expression;
(2) Open-field control (CTR-OF): the animals were euthanized after four expositions to the open-field without any objects. The goal of this group was to control IEGs expression as consequence of exploring a familiar arena;
(3) Open-field + object control (CRT-OF + Obj): the rats were exposed to the empty apparatus during the habituation and two training sessions, and to a test session in the open-field with one object placed in a random location. The goal of this group was to control IEGs expression as result from sensory activity. Only one object was placed in order to avoid mnemonic processes (comparing objects in different locations);
(4) OR: the animals were submitted to the habituation session, two training sessions with four copies of one object in the same positions on both trials and a test session with two copies of objects used in the training sessions and two novel objects (in the same locations). The exploration rates in the test session were expected to be higher for novel objects than for familiar objects;
(5) ELM task: this task consisted of a habituation session, two training trials and a test session. In the first training, the animals were placed in the open-field with four copies of an object in a certain spatial configuration. After 1 h, in the second training, they were submitted to a set of four copies of another object in a different spatial configuration. Spatial configurations were random across subjects. In the test session, two copies of the objects from each training session were presented. One copy from the first trial was placed in the same location (old familiar-stationary) and the other copy was placed in a new location (old familiar-displaced). The same procedure was conducted with the recent familiar objects (those presented in the second training). According to Kart-Teke et al. (2006, 2007), the animals were expected to explore the recent displaced object more than the recent stationary object, and the opposite pattern is expected regarding the old objects. This inverse pattern would indicate that the rats integrated the “what-where-when” components of the ELM, contrary to similar ELM tasks (Dere et al., 2005a; Barbosa et al., 2010).

### Immunohistochemistry

Sixty minutes after the last behavioral procedure, rats were deeply anesthetized with intraperitoneal injection of the sodium thiopental (40 mg/kg) and perfused transcardially with 200 ml of phosphate-buffered saline (PBS), pH 7.4, containing 500 IU heparin (Liquemin, Roche, Brazil), followed by 300 ml of 4.0% paraformaldehyde in 0.1 M phosphate buffer, pH 7.4 (fixative solution). This interval was required aiming the expression peaks of Zif-268 and c-Fos (Bisler et al., 2002). The brains were removed from the skull, post-fixed in fixative solution for 2–4 h, and transferred to a solution containing sucrose 30% in 0.1 M PBS, pH 7.4. Each brain was serially cut in the coronal plane into 30-μm thick sections with a cryostat microtome (Leica, Germany) at a temperature of −20°C. The sections were placed sequentially in five compartments (one section per compartment), with the distance between one section and the next in the same compartment being approximately 150 μm. All sections were stored in antifreeze solution. For the detection of c-Fos and Zif-268, free-floating sections were incubated for 18–24 h with a monoclonal primary antibody raised in rabbits (Zif-268 antibody, SantaCruz Biotechnology, and c-Fos antibody, Oncogene Science, Cambridge, UK; both diluted 1:1000), containing 2% goat normal serum (Sigma Chemical Company), diluted in 0.3% Triton X-100 (ICN Biomedicals), and 0.1 M phosphate buffer, pH 7.4. Afterward, the sections were incubated with the biotinylated secondary anti-rabbit antibody raised in goat (1:1000; Jackson), also diluted in 0.3% Triton X-100 and 0.1 M phosphate buffer, pH 7.4. This procedure lasted 2 h and was performed at room temperature. Shortly after, the sections were washed and incubated in 2% avidin-biotin-peroxidase solution (ABC Elite kit, Vector Labs, Burlingame, CA, USA) for 90 min. The reaction was developed by the addition of 2.5% diaminobenzidine tetrahydrochloride (Sigma, St. Louis, MO, USA) and 0.01% H_2_O_2_ in 0.1 M phosphate buffer, pH 7.4. The sections were washed (four times, 5 min) with 0.1 M phosphate buffer, pH 7.4, between each step and at the end of the procedure. Afterward, the sections were dried, dehydrated in a graded alcohol series, cleared in xylene, and coverslipped with Entellan (Merck).

### Image analyses and cell count

Sections were examined under brightfield illumination (Olympus Microscope, BX-41). Images were captured using a CCD camera (Nikon, DXM-1200) and the locations of areas were determined using the atlas of Paxinos and Watson (2007). The cell count was performed manually in three sections per animal, through Image J software (1.46i, NIH) and the mean count was calculated and used in the analysis. Positive c-Fos and Zif-268 cells were counted in areas of the PRh, entorhinal cortex (ERH), dorsal hippocampal subregions (CA1, CA3, and DG), HP (calculated by the sum of the values of the three subregions), mPFC, and primary visual cortex (V1). The experimenter was blinded to experimental groups during counting. The number of cells for each brain area was normalized by mean values of the control group (CTR-HC).

### Data collection and analysis

The parameters analyzed in the open-field were total distance traveled and time and exploration ratio of objects. The analyses were made by an experimenter blind to groups, who used keys to score exploration when the animals approached an object and had physical contact with it, either with the forepaws and/or snouts. The exploration ratio was the time exploring an object/total time exploring all objects. Object exploration ratios were calculated for novel objects in the OR task and displaced, stationary, old familiar, and recent familiar objects in the ELM task. The Kolmogorov–Smirnov test indicated normal data distribution. One-way ANOVAs were performed for total distance traveled and total time exploring the objects in the last session to analyze possible difference in motivation between groups. *A priori* planned dependent *t*-tests were used to compare novel and familiar objects in the OR group, considering the initial 2 min of the test session as suggested by others as the optimal time window analyses (Dix and Aggleton, 1999; Mumby et al., 2002). In the ELM task, displaced old familiar × stationary old familiar, and displaced recent familiar × stationary recent familiar were compared by dependent *t*-tests considering the total time of the test session (5 min). One-way ANOVAs were used for comparison of the number of positive Zif-268 or c-Fos neurons between groups in each brain area. *Post hoc* analysis was conducted with the Tukey–Kramer’s test. Pearson’s test was used to investigate correlations (*r*) for the cell count values among areas, as well as between behavioral parameters and cell count values in each area. Only areas that showed significant increase in the IEGs expression were included in the correlation analysis. Results were expressed as mean ± SEM. In all statistical tests, effects were considered significant when *p* < 0.05.

## Results

### Behavioral tasks

One-way ANOVA showed no significant differences in the total distance traveled in the habituation [*F*(3,27) = 0.84, *p* = 0.483], training 1 [*F*(3,27) = 1.64, *p* = 0.203], and training 2 [*F*(3,27) = 0.79, *p* = 0.509] sessions. Significant differences were detected in the total distance traveled in the test session [*F*(3,27) = 3.39, *p* = 0.032]. The Tukey–Kramer’s *post hoc* revealed increased distance traveled by ELM group when compared to the CTR-OF group in the test session (*p* = 0.028; see Table 1). However, in this session, no differences were found in the total time of object exploration [*F*(2,20) = 0.76, *p* = 0.481], suggesting that all groups exposed to objects (CTR-OF + Obj, OR and ELM) present similar motivation to explore (see Table 1).

**Table 1:** **Total distance traveled (m) and total object exploration time (s) by groups in each session (mean ± SEM)**.

	CTR-OF	CTR-OF + Obj	OR	ELM
**TOTAL DISTANCE (m)**				
Habituation	27.82 ± 2.30	30.44 ± 3.49	29.43 ± 2.85	33.32 ± 2.22
Training session-1	14.44 ± 1.70	16.28 ± 3.18	16.08 ± 2.60	20.96 ± 1.92
Training session-2	9.44 ± 2.27	11.91 ± 2.22	12.71 ± 0.98	13.35 ± 2.15
Test	6.86 ± 1.62	13.08 ± 1.65	10.63 ± 2.16	13.93 ± 1.43*
**TOTAL EXPLORATION (s)**				
Training session-1	–	–	52.08 ± 12.18	64.69 ± 9.86
Training session-2	–	–	42.55 ± 10.34	45.03 ± 7.74
Test	–	33.32 ± 7.16	48.35 ± 12.45	48.88 ± 6.98

CTR-OF, open-field control; CRT-OF + Obj, open-field + object control; OR, object recognition task; and ELM, episodic-like memory task. *p < 0.05 compared to CTR-OF (one-way ANOVA and Tukey–Kramer’s post hoc).

**Figure 2:**
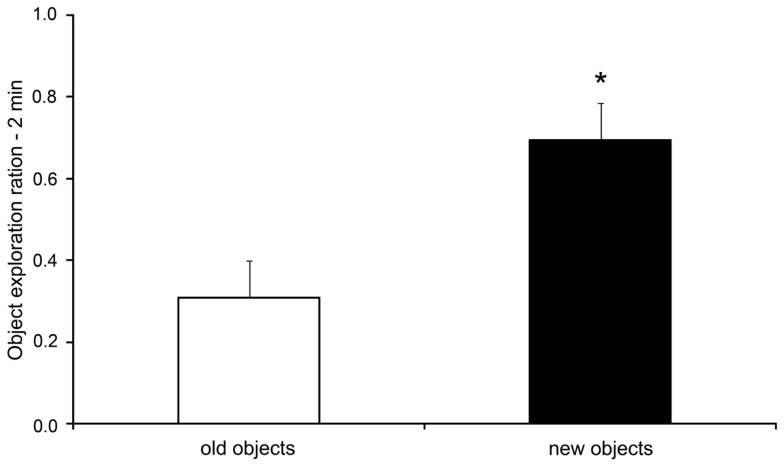
**Exploration ratios (mean + SEM) within the initial 2 min of the test session of the object recognition task**. *p < 0.05 compared to old objects exploration (paired samples*t*-test).

As expected, the rats submitted to the OR task presented an increase in the exploration ratio of novel objects when compared to exploration of the old object in the test session [*t*(7) = 2.45; *p* = 0.044, two-tailed *t*-test for paired samples], as shown in Figure 2. During the training sessions 1 and 2, there were no differences in the exploration ratio of the objects [*F*(3,21) = 1.63, *p* = 0.212 and *F*(3,21) = 1.59, *p* = 0.220, respectively, data not shown].

**Figure 3:**
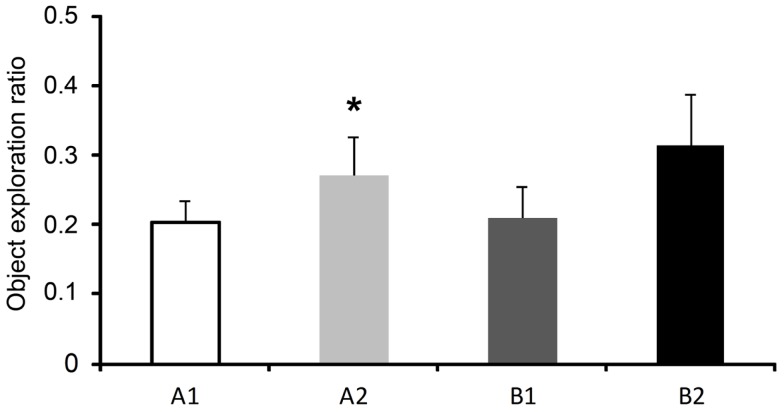
**Exploration ratios (mean ± SEM) within 5 min of the test session of the episodic-like memory (ELM) task**. Objects: A1, stationary old familiar; A2, displaced old familiar; B1, stationary recent familiar; and B2, displaced recent familiar. *p < 0.05 compared to A1 (paired two-tailed*t*-test).

During the training sessions 1 and 2 of the ELM task there were no differences in the exploration ratio of similar objects [*F*(3,21) = 2.57, *p* = 0.132 and *F*(3,21) = 1.16, *p* = 0.339, respectively]. In the test session, the rats presented increased exploration ratio of the displaced old familiar object compared to the stationary old familiar object [*t*(8) = 2.86; *p* = 0.021]. No difference was found when the exploration ratios of the recent familiar objects were compared [*t*(8) = 0.98; *p* = 0.354], as shown in Figure 3.

### c-Fos expression

**Figure 4:**
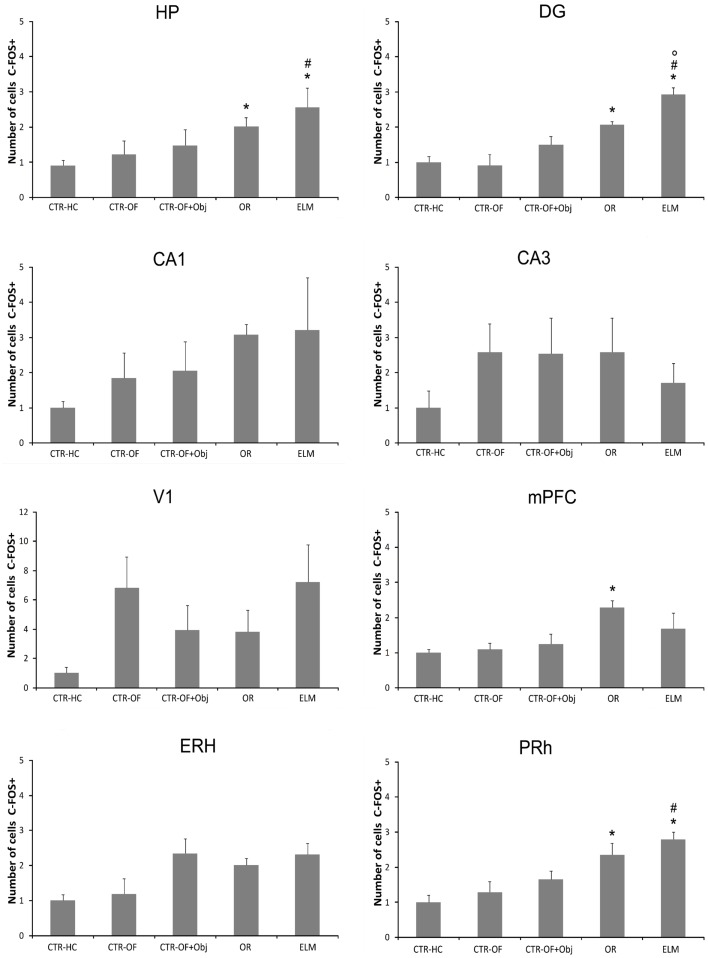
**Expression of c-Fos in different brain areas (HP, hippocampus; DG, dentate gyrus; CA1; CA3; mPFC, medial prefrontal cortex; V1, visual area 1; ERH, entorhinal cortex; PRh, perirhinal cortex) for home cage control (CTR-HC), open-field control (CTR-OF), open-field + object control (CRT-OF + Obj), object recognition (OR), and episodic-like memory (ELM) groups**. The normalized number of cells is expressed as the mean ± SEM. *p < 0.05 compared to CTR-HC and CTR-OF; ^#^*p* < 0.05 compared to CTR-OF + Obj; °*p* < 0.05 compared to OR (one-way ANOVA followed by Tukey’s test).

**Figure 5:**
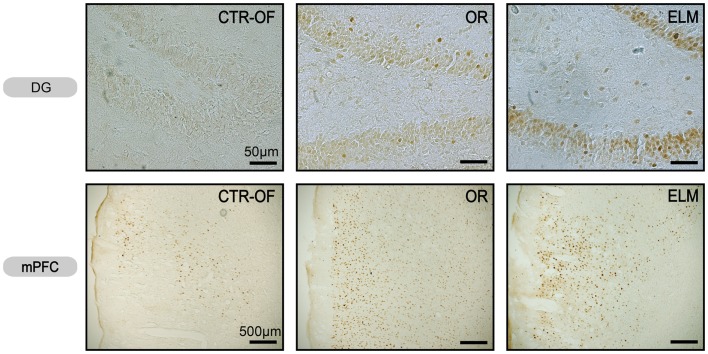
**Representative images of c-Fos expression in dentate gyrus (DG) and medial prefrontal cortex (mPFC) from subjects of control open-field + object (CTRL-OF + Obj), novel object recognition (OR), and episodic-like memory (ELM) task groups**.

For the number of c-Fos-positive cells, one way ANOVA revealed significant differences between groups for HP [*F*(4,34) = 5.96, *p* = 0.001], DG [*F*(4,34) = 16.51, *p* < 0.001], mPFC [*F*(4,34) = 3.88, *p* = 0.011], ERH [*F*(4,34) = 3.37, *p* = 0.020], and PRh [*F*(4,34) = 8.32, *p* < 0.001]. No differences were detected in the CA1 [*F*(4,34) = 1.48, *p* = 0.228], CA3 [*F*(4,34) = 1.45, *p* = 0.238], and V1 [*F*(4,34) = 1.69, *p* = 0.174]. *Post hoc* analysis revealed increased number of c-Fos-positive cells in the OR group compared to CTR-HC and CTR-OF in HP, DG, mPFC, and PRh. The number of c-Fos-positive-cells was also increased in ELM when compared to CTR-HC, CTR-OF, and CTR-OF + Obj in HP, DG, and PRh. ELM also showed increased number of c-Fos positive cells when compared to OR in DG. Mean results for counts in all groups are shown in Figure 4, and representative images of some areas are displayed in Figure 5.

### Zif-268 expression

**Figure 6:**
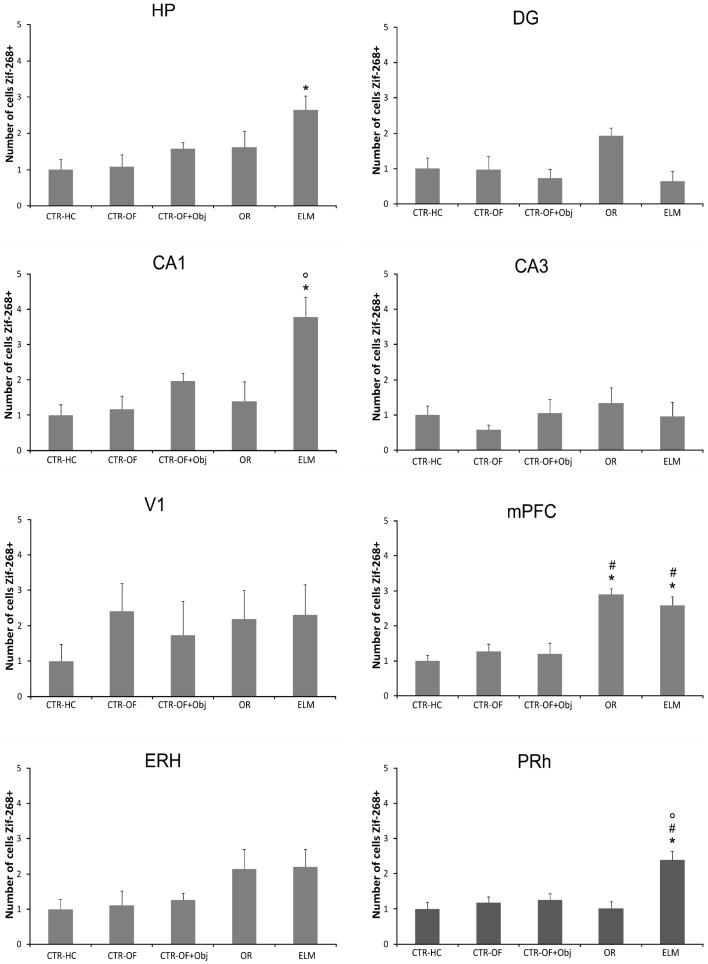
**Expression of Zif-268 in different brain areas (HP, hippocampus; DG, dentate gyrus; CA1; CA3; mPFC, medial prefrontal cortex; V1, visual area 1; ERH, entorhinal cortex; PRh, perirhinal cortex) for home cage control (CTR-HC), open-field control (CTR-OF), open-field + object control (CRT-OF + Obj), object recognition (OR) episodic-like memory (ELM) task groups**. The normalized number of cells is expressed as the mean ± SEM. *p < 0.05 compared to CTR-HC and CTR-OF; ^#^*p* < 0.05 compared to CTR-OF + Obj; °*p* < 0.05 compared to OR (one-way ANOVA followed by Tukey’s test).

**Figure 7:**
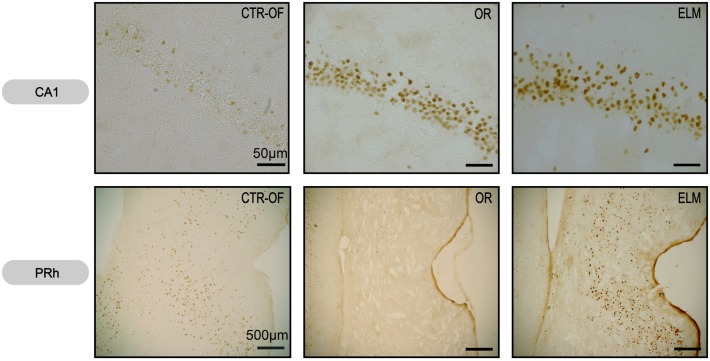
**Representative images of Zif-268 expression in CA1 hippocampal subregion and perirhinal cortex (PRh) from subjects of control open-field + object (CTRL-OF + Obj), novel object recognition (OR), and episodic-like memory (ELM) task groups**.

For the number of Zif-268-positive cells, one way ANOVA revealed significant differences between groups in HP [*F*(4,34) = 3.62, *p* = 0.005], DG [*F*(4,34) = 2.94, *p* = 0.034], CA1 [*F*(4,34) = 6.57, *p* < 0.001], mPFC [*F*(4,34) = 21.19, *p* < 0.001], and PRh [*F*(4,34) = 8.39, *p* < 0.001]. No differences were detected in the CA3 [*F*(4,34) = 0.59, *p* = 0.669], V1 [*F*(4,34) = 1.08, *p* = 0.380], and ERH [*F*(4,34) = 2.22, *p* = 0.087]. *Post hoc* analysis revealed increased number of Zif-268-positive cells in OR group when compared to CTR-HC, CTR-OF, and CTR-OF + Obj in the mPFC. The analysis also showed increased number of Zif-268-positive cells in mPFC and PRH of the ELM group when compared to the three other groups. In addition, increased values were found in HP of this group compared to CTR-HC and CTR-OF. ELM also showed increased values when compared to CTR-OF + Obj in mPFC and PRh, as well as compared to OR in PRh and CA1. Although one-way ANOVA revealed significant differences between groups in the DG, differences were not detected by the Tukey–Kramer’s *post hoc* test. Mean results for counts in all groups are shown in Figure 6, and representative images of some areas are displayed in Figure 7.

### Correlations

Pearson’s correlation tests were applied to the number of c-Fos and Zif-268 positive neurons in each area against the exploration rate of objects for each task. We also ran correlations of the number of c-Fos and Zif-268 positive neurons among areas that showed increase in the IEGs expression in the previous analyses (CA1, DG, PRh, and mPFC) after OR and ELM tasks. These correlations are shown in Table 2. Values (*r*; *p*) for non-significant correlations were omitted. As shown in the table, all coefficients (*r*) were above 0.6, indicating large effect sizes for the correlations found.

**Table 2:** **Correlations to the number of c-Fos and Zif-268 positive neurons in the analyzed areas against the exploration rate of objects during novel object recognition (OR) or episodic-like memory (ELM) task**.

	OR	ELM
	Familiar objectsexploration ratio (2 min)	Displaced familiar objectexploration ratio (5 min)
c-Fos		PRh (0.71; 0.032)
Zif-268	PRh (0.79; 0.020)	DG (0.66; 0.050)*
		PRh (0.79; 0.010)
		DG-PRh (0.71; 0.02)^a^

Areas: hippocampal area (dentate gyrus – DG), and perirhinal cortex (PRh). Significant correlations (Pearson’s correlation – r) when p < 0.05. *Marginally significant effects.

*^a^Significant correlations shown by Pearson’s test (r; p) to the number of Zif-268 positive neurons between areas after episodic-like memory (ELM) task*.

## Discussion

We evaluated whether OR tasks with different cognitive demands produce a varied pattern of expression of the IEGs c-Fos and Zif-268 in the medial temporal lobe structures and in the mPFC. We found a greater c-Fos and Zif-268 expression in the dorsal HP, perirhinal, and mPFC in the ELM and OR groups when compared to the different control groups. This indicates that the activation of these structures is neither a consequence of exploration of a familiar arena nor due to the process of object exploration. More importantly, we found some differences between the activation of neural networks induced by the ELM and OR protocols. Specifically, the first one promoted increased c-Fos expression in the DG and increased Zif-268 expression in the PRh and CA1 compared to the OR, indicating a greater involvement of these regions in the retrieval of the task with spatial cognitive demand (as discussed in detail below). As commented in the Section “Introduction”, increases in IEGs expression in the hippocampal regions and in the prefrontal cortex after the ELM task were expected. However, unexpectedly, an increase in the Zif-268 expression in the PRh was also found.

As expected to the OR task, rats explored more the new objects when compared to the old objects, indicating recognition memory (see Figure 2). It has been suggested that the recognition memory is supported by two distinctive cognitive processes: familiarity and recollection (Brown and Aggleton, 2001; Eichenbaum et al., 2007). In the OR task, rats could use only familiarity to discriminate the objects. On the other hand, in the ELM task rats had to use the recollection process to discriminate the order of presentation and positions of the objects (Dere et al., 2006; Kart-Teke et al., 2006). In this task, we expected that rats would spend more time exploring the displaced recent object when compared to the stationary recent object, and the opposite pattern is expected to the old familiar objects (Kart-Teke et al., 2006, 2007; Li and Chao, 2008). Kart-Teke et al. (2006) suggested that this inverse pattern would be indicative that Wistar rats created an integrative what-where-when memory. However, in the present study, we did not found the same pattern of results. Rats spent more time exploring the displaced when compared to the stationary old familiar object and did not discriminate the recent familiar objects. Therefore, we cannot assume that the rats recalled a what-where-when memory. It is important to note, however, that in the present study we used a 24-h interval and not a 1-h delay as used by Kart-Teke and colleagues. This variation in the protocol could explain these different results. We have decided to use this interval to avoid a possible ceiling effect in the IEGs expression, as well to separate the retrieval mnemonic process from the acquisition and consolidation processes (Bisler et al., 2002; Barbosa et al., 2010). Regardless, the present results clearly show that rats used associative recognition memory, because they could discriminate spatially the old familiar objects, similarly to the object-in-place task used by others (Dix and Aggleton, 1999; Barker et al., 2007). In addition, it has been demonstrated that the OR and object-in-place tasks are supported by different neural substrates (Mumby et al., 2002; Barker et al., 2007; Barker and Warburton, 2011), and this finding was also reported here regarding OR and ELM tasks, as discussed below. Thus, in the present study, we can assume that rats accessed at least what-where aspects of the ELM. For this reason we discuss the outcome of the ELM task in terms of what-where memory or spatial memory.

Regarding c-Fos expression (Figures 4 and 5), we found a greater activation of the DG in the ELM task when compared to all other groups; including the novel OR task. The DG has been implicated in the detection of spatial novelty (Kesner, 2007; Leutgeb et al., 2007; Hunsaker and Kesner, 2008; Hunsaker et al., 2008), and some theoretical authors have suggested that this structure is essential to the spatial pattern separation (McClelland et al., 1995; Norman and O’Reilly, 2003; Treves et al., 2008). Accordingly, we have shown that temporary inactivation of this region can impair the what-where acquisition and/or consolidation processes (Barbosa et al., 2012). Muscimol injection before the first training session produced impairment in the spatial novelty detection, but not in the temporal order memory. Therefore, the increase in c-Fos expression seems in agreement with previous studies. However, it is important to note that we accessed IEG expression after the test session and therefore, in the present work, we analyzed a different mnemonic process. More studies are necessary to verify a causal relation between this HP region and retrieval of spatial memory.

Contrary to previous studies (Albasser et al., 2010, 2013; Rinaldi et al., 2010; Castilla-Ortega et al., 2012), we did not found any difference in the c-Fos expression in the dorsal CA1 and CA3 subregions. Castilla-Ortega et al. (2012) described an increase in c-Fos expression in the CA1 subregion after ELM task, but no alterations in CA3 compared to the home cage group. It is important to point out that this previous study had a 90-min delay before retrieval while in the present study we used a 24-h delay between the second sample and the test session. More importantly, the mice in that previous study did not discriminate the displaced familiar object (that was the only displaced object in that study). Further, we also added other control groups beyond the home cage, as a way to control other possible variables that could interfere with IEGs expression as exploratory activity in the open-field and object exploration *per se*. Albasser and collaborators (Albasser et al., 2010; Aggleton et al., 2012) found a greater c-Fos expression in the dorsal CA3 subregion in rats exposed to novel objects when compared to rats exposed to familiar objects. The behavioral protocol in this case is very dissimilar from the present one, since rats were exposed in multiple trials to novel or familiar objects in a bow-tie-shaped maze. However, hippocampal lesion did not impair novelty object discrimination in that task (Albasser et al., 2013).

No difference was detected in the c-Fos expression in the lateral entorhinal between the groups. This medial temporal lobe region has been implicated in item novelty detection. Indeed, Hunsaker et al. (2013) showed that excitotoxic lesion of the lateral ERH (but not the medial portion) disrupted novel OR memory. Interestingly, the medial entorhinal lesion impaired contextual novelty detection, but not the detection of a novel item. However, we did not found any change in the IEGs expression analyzed here in this area. It is important to point out that we found a tendency (*p* = 0.08) toward an increase in Zif-268 expression in this region. Thus, with a larger sample size we would probably find a significant difference. More studies are needed to evaluate better the role of the lateral ERH in the retrieval of recognition memory.

Regarding perirhinal c-Fos expression, no difference was detected between OR and ELM groups, but both had increased number of positive cells relative to the control groups. These two groups were exposed to four objects in the test session, which was not the case of the home cage and open field groups (not exposed to any objects) and the open field plus one object group (explored only one object). Several studies have proposed that this region is fundamental to item novelty detection (Winters and Bussey, 2005a,b; Barker and Warburton, 2008), and more recently, lesion studies indicated also a role in the object-in-place task (Barker et al., 2007; Barker and Warburton, 2011). Therefore, this region seems to be essential in both OR tasks and the present results corroborate this idea.

Although lesion studies indicate that the mPFC is not involved in the detection of a novel item (Barker et al., 2007; Barker and Warburton, 2011; DeVito and Eichenbaum, 2011), some authors found an increase in the c-Fos expression after rodents were exposed to an OR memory task (Rinaldi et al., 2010; Castilla-Ortega et al., 2012). We also found increased activation of this area in the OR group when compared to the control groups. However, no difference was found between the OR and ELM groups, corroborating the previous finding by Castilla-Ortega et al. (2012). On the other hand, studies with lesions have showed a role of this region in the object-in-place task, as well as an interaction of the mPFC with the HP (Barker and Warburton, 2011). Kim et al. (2011) showed that in object-in-place learning “CA1-mPFC coherence in theta oscillation was maximal before entering a critical place for decision making,” which indicates an integrative role of these neural regions. Interestingly, we found a greater expression of Zif-268 in the OR and ELM relative to all the control groups. As one can see, only in these two tasks rats had to make some decision. Thus it seems quite possible that the mPFC is involved in this cognitive process, although lesion studies indicate that, at least in the item recognition, it is not always determinant to the output behavior. Another possible explanation to the involvement of this region is related to the previously reported role of mPFC in the long term memory consolidation and recall processes (Frankland and Bontempi, 2005; Leon et al., 2010).

Immediate-early genes expression data indicated that there were different neural networks involved considering the activation pattern of OR and ELM groups. Thus, we investigated possible co-activations between structures as evaluated by IEGs expression, as well as correlation between activation of structures and output behavior (Table 2) in groups that went through OR and ELM tasks. In the ELM task, a positive correlation between the PRh c-Fos expression and the displaced old familiar object exploration ratio was found. This result is in agreement with lesion studies indicating a role of this structure in the object-in-place task (Barker et al., 2007; Barker and Warburton, 2011). It is important to note, however, that these are correlation findings which do not imply causal relations between IEGs expressions and the behavioral outcomes.

There are few studies evaluating the involvement of Zif-268 expression in recognition memory. It is known that the *zif-268* gene expression is involved in the consolidation of item and memory location of objects (Bozon et al., 2003a,b). Soulé et al. (2008) showed that the *zif-268* expression was elevated in the DG after rats were exposed to an object location task. As mentioned above, we found an increase in c-Fos expression in the DG only after what-where memory retrieval. This group also had the greatest Zif-268 expression in the dorsal CA1 subregion (Figures 6 and 7). In this respect, we have recently shown that temporary inactivation of CA1 subregion before training impairs both temporal and spatial components of the ELM, which is in accordance with the present results (Barbosa et al., 2012).

The ELM rats had also the greatest Zif-268 expression in the PRh (Figures 6 and 7). As commented before, this region has been pointed as critical to object-in-place task (Brown and Aggleton, 2001; Aggleton et al., 2012). Again our results are in agreement with these lesion studies. Additionally, a positive correlation between the perirhinal Zif-268 expression and the familiar objects exploration ratio was detected (Table 2). Interestingly, to our knowledge, this is the first study to show a positive correlation between Zif-268 expression and time that rats spent exploring familiar objects. Previous studies showed a negative correlation between perirhinal c-Fos activity and exploration of familiar stimuli (Wan et al., 1999, 2001), which was not detected in the present work.

We also found different neural networks co-activated in the ELM group (Table 2), regarding Zif-268 expression. Interestingly, PRh activity was correlated with DG. The co-activation of the PRh with DG corroborates studies indicating that both the HP and this medial temporal lobe region are recruited in the object-in-place task (Barker and Warburton, 2011). Moreover, our results suggest that CA1 does not co-activate with the PRh during this task. In this context, it is known that the PRh cortex has direct projections to the CA1 subfield, and indirect connections, via lateral ERH, to DG and CA3 (van Strien et al., 2009; Kealy and Commins, 2011). Probably the most important finding here was the positive correlation between both DG and PRh Zif-268 expressions and the displaced familiar object exploration ratio. Interestingly, these results seem to corroborate the Binding of Items and Context (BIC) model that proposes that item memory (what) is processed preferentially in the PRh cortex (and the lateral ERH) and that the contextual information is processed initially in the medial ERH, while item and contextual elements would be bound together in the HP (Eichenbaum et al., 2007; Hunsaker et al., 2013). Indeed, as mentioned, we found co-activation of the PRh and DG in the ELM task used here. In addition, both neural areas positively correlated with the behavioral output, although the significance was marginal in the case of the DG.

It is important to note that the differential IEGs expression across the groups did not follow the same pattern when c-Fos and Zif-268 are considered. In this respect, although these IEGs are both involved in plastic processes, they have different biochemical routes (Bisler et al., 2002; Davis et al., 2003) and probably different functions. Additionally, while c-Fos has mostly been implicated in the exposure to novel stimuli, or as a consequence of stimulation after sensory deprivation, Zif-268 expression is probably related to persistent synaptic stimulation (see Chaudhuri et al., 2000). Indeed, it has been shown that Zif-268 is required for different types of learning, including OR-based tasks (Jones et al., 2001; Bozon et al., 2002). The implication for the present results is that the increased c-Fos expression after both OR and ELM tasks (that were, in general, similar across areas) would be related to the novelty present in the test situation compared to previous sessions. Conversely, the increased Zif-268 expression could reflect the activation of the structures engaged in plastic mechanisms related to the retrieval of the tasks. Accordingly, areas suggested to be more implicated in consolidation of spatial and temporal aspects of an event rather than standard OR tasks were activated after ELM, but not OR task.

Finally, it is important to point out that the differences found in IEGs expression cannot be explained by a general activation, because we did not detected any differences in the expression of c-Fos and Zif-268 in the control area V1. In addition, the groups did not presented differences in the total amount of object exploration in the last behavioral session. Thus, it is unlikely that the pattern of IEGs expression described here is a consequence of motor and/or sensory activity.

In conclusion, the present data show increased IEGs expression in brain areas related to memory processes due to retrieval of OR-based tasks, but not as a consequence of general behavioral procedures. Also, the pattern of activation was different in tasks with different cognitive demands. Taken together, the analyses of c-Fos and Zif-268 expressions suggest the activation of CA1 and DG hippocampal subregions, as well as PRh after what-where memory retrieval, while the standard OR task seems to involve mPFC, DG, and PRh areas. Accordingly, correlation tests suggest the engagement of different neural networks in the OR tasks used. Specifically, perirhinal-hippocampal co-activation was detected after the what-where memory retrieval, which correlated with the respective behavioral outcome. These findings can be helpful in the understanding of the neural networks underlying memory tasks with different cognitive demands.

## Conflict of Interest Statement

The authors declare that the research was conducted in the absence of any commercial or financial relationships that could be construed as a potential conflict of interest.
